# Cellular prion protein is present in mitochondria of healthy mice

**DOI:** 10.1038/srep41556

**Published:** 2017-02-02

**Authors:** Robert Faris, Roger A. Moore, Anne Ward, Brent Race, David W. Dorward, Jason R. Hollister, Elizabeth R. Fischer, Suzette A. Priola

**Affiliations:** 1 Laboratory of Persistent Viral Diseases, Rocky Mountain Laboratories, National Institute of Allergy and Infectious Diseases, Hamilton, National Institutes of Health, Montana 59840, USA; 2 Research Technologies Branch, Rocky Mountain Laboratories, National Institute of Allergy and Infectious Diseases, National Institutes of Health, Hamilton, Montana 59840, USA.

## Abstract

Cellular prion protein (PrP^C^) is a mammalian glycoprotein which is usually found anchored to the plasma membrane via a glycophosphatidylinositol (GPI) anchor. PrP^C^ misfolds to a pathogenic isoform PrP^Sc^, the causative agent of neurodegenerative prion diseases. The precise function of PrP^C^ remains elusive but may depend upon its cellular localization. Here we show that PrP^C^ is present in brain mitochondria from 6–12 week old wild-type and transgenic mice in the absence of disease. Mitochondrial PrP^C^ was fully processed with mature N-linked glycans and did not require the GPI anchor for localization. Protease treatment of purified mitochondria suggested that mitochondrial PrP^C^ exists as a transmembrane isoform with the C-terminus facing the mitochondrial matrix and the N-terminus facing the intermembrane space. Taken together, our data suggest that PrP^C^ can be found in mitochondria in the absence of disease, old age, mutation, or overexpression and that PrP^C^ may affect mitochondrial function.

Cellular prion protein (PrP^C^) can pathogenically misfold into PrP^Sc^ which is the main component of the infectious prions which cause prion diseases in humans and other mammals^1^. Creutzfeldt-Jakob disease (CJD) is the most prevalent of the human prion diseases, occurring with sporadic, genetic and infectious etiologies^2^. Normally folded PrP^C^ is a ubiquitous, non-pathogenic glycoprotein most often found bound to the outer leaflet of the plasma membrane by a glycophosphatidylinositol (GPI) anchor. The potential physiological functions of PrP^C^ have been studied for over three decades, yet much debate persists regarding normal PrP^C^ function and how it might impact prion pathogenesis. The cellular functions proposed to date for PrP^C^ include regulation of copper metabolism, cell migration, cell differentiation, long term potentiation, cell survival, and inhibition of Bax mediated apoptosis^3^,^4^,^5^,^6^,^7^,^8^.

Since the precise physiological functions of PrP^C^ remain enigmatic, in depth examination of the subcellular localization of PrP^C^ in healthy animals could reveal mechanistic information relevant to prion disease. PrP^C^ has been observed under certain conditions in a variety of subcellular locations other than the plasma membrane including the cytoplasm, nucleus, neuronal synapse, and mitochondria of 520 day old transgenic PrP^C^-overexpressing mice^9^,^10^,^11^,^12^,^13^,^14^,^15^,^16^. However, whether normal mammalian derived PrP^C^ from non-transgenic animals can be consistently found in subcellular locations besides the plasma membrane, is not clear. Mutation and/or overexpression of PrP^C^ can induce its retention in the endoplasmic reticulum (ER) and Golgi^17^,^18^. In addition to its normal presence on the plasma membrane, PrP^C^ can be transiently detected in the ER and Golgi, presumably as it undergoes modification of its N-linked glycans, addition of the GPI anchor, and packaging into secretory vesicles. Transmembrane forms of PrP^C^ have been reported in both ER and Golgi^18^,^19^,^20^ and an early study suggested that these isoforms result in neurodegenerative disease^21^. Thus, altered PrP^C^ membrane topology may be an important determinant in the processes underlying the neurodegeneration observed in prion disease.

Neuronal mitochondria play a critical role in maintaining cellular homeostasis, modulating reactive species, providing energy in the form of ATP by oxidative phosphorylation, and regulating various forms of programmed cell death among a plethora of other functions^22^,^23^. Bioenergetic gradients across the inner mitochondrial membrane drive production of most of the cellular ATP required for cellular survival and disruption of mitochondrial bioenergetics is implicated in most, if not all neurodegenerative disorders including amyotrophic lateral sclerosis (ALS), Parkinson’s, Alzheimer’s, and Huntington’s diseases^24^,^25^. Mitochondrial dysfunction has also been observed in mouse and hamster models of prion infection. An early study by Aiken and colleagues suggested that the scrapie agent, or prion, was present in brain mitochondria from prion-infected hamsters^26^ while Siskova and colleagues proposed that mitochondrial dysfunction underlies prion-associated synaptic degeneration^27^. Choi *et al*. found that prion infected hamsters had decreased manganese superoxide-dismutase, cytochrome c oxidase, and ATPase activity suggesting that mitochondrial dysfunction was occurring at the clinical stage of disease^28^.

Evidence for a physical interaction between PrP^C^ and mitochondria or confirmation of a lack thereof would help provide further insight into how PrP may affect mitochondrial function. However, mitochondrial localization of PrP^C^ has only been reported in 520 day old transgenic mice^16^ and it is unclear whether its presence in mitochondria is an artifact of PrP^C^ overexpression. While PrP^C^ overexpression correlates with shorter disease incubation times in transgenic mouse models of prion disease^29^, transgenic mice lacking PrP^C^ (PrP^*0*/*0*^ mice) are immune to prion infection^30^ and appear developmentally normal^30^,^31^. Although respiration activities in brain mitochondria from PrP^*0*/*0*^ mice have been reported as normal^32^, studies in adult animals have demonstrated that the absence of PrP^C^ results in deficits in long-term potentiation, memory and learning problems, increased levels of oxidative stress, and abnormalities in mitochondrial morphology^4^,^31^,^33^,^34^. Thus, some of these deficits in PrP^*0*/*0*^ mice could indicate that PrP^C^ normally participates in mitochondrial physiology within the brain.

In this study we show that PrP^C^ can be found in mitochondria from the brains of healthy, young wild-type C57BL/6 mice, two PrP^C^-overexpressing transgenic mouse lines, and transgenic mice expressing low levels of PrP^C^ without the GPI anchor. The vast majority of mitochondria-localized PrP^C^ was associated with the mitoplast which consists of the inner mitochondrial membrane and matrix. PrP^C^ associated with the mitoplast was glycosylated and appeared to span the inner mitochondrial membrane with the C-terminus facing the matrix and the N-terminus oriented toward the intermembrane space. Cumulatively, our results offer compelling evidence for the mitochondrial localization of PrP^C^ under homeostatic conditions and are consistent with a role for PrP^C^ in mitochondrial function.

## Results

### PrP^C^ in brain mitochondria isolated from both wild-type C57BL/6 and transgenic mouse lines

Previous reports of PrP^C^ detection in mitochondria utilized aged transgenic animals overexpressing PrP^C^ raising the possibility that localization of PrP^C^ to the mitochondrion was an artifact of overexpression and/or age^16^. To determine whether PrP^C^ localization to the mitochondrion was dependent on PrP^C^ overexpression or age, we utilized 6–12 week old wild-type C57BL/6 mice as well as transgenic mice that overexpress PrP^C^ (RM and Tg3F4). We also used 6–12 week old Tg44 transgenic mice which underexpress GPI-anchorless PrP^C^^35^,^36^ to determine whether the GPI anchor was necessary for mitochondrial localization. Mitochondria were isolated from fresh brain tissue of C57BL/6, RM, Tg3F4, and Tg44 mice using the MACS isolation procedure as described in methods. To confirm that the isolation procedure yielded intact mitochondria, a sample of MACS isolated mitochondria from C57BL/6 mice was prepared for Cryo-EM and immunogold labeled using an antibody against the inner mitochondrial membrane protein COXIV. Nearly all observable structures stained for COXIV suggesting a highly enriched mitochondrial preparation (Supplementary Fig. S1).

**Figure 1:**
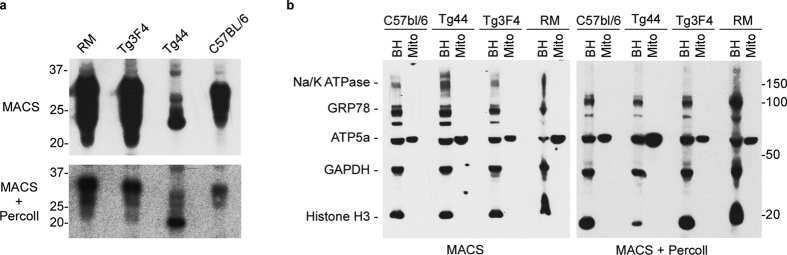
PrP^C^ is present in mitochondria isolated from the brains of wild-type C57BL/6 and transgenic mice. (**a**) Top panel: PrP^C^ is detectable in MACS purified mitochondria isolated from brain tissue of wild-type C57Bl/6 mice, transgenic mice overexpressing human PrP^C^ (RM), overexpressing mouse PrP^C^ containing the 3F4 epitope (Tg3F4), or mice underexpressing mouse PrP^C^ without the GPI anchor (Tg44). Bottom panel: PrP^C^ is detectable in highly purified MACS + Percoll mitochondrial preparations from which MAM have been removed. Each lane was loaded with 10 μg of MACS purified mitochondrial lysate or MACS + Percoll lysate. The rabbit monoclonal antibody EP1802Y, which recognizes a C-terminal epitope of PrP^C^, was used for detection. Molecular mass markers in kilodaltons are shown on the left. Each panel represents an individual blot. Blots were cropped for presentation purposes and to remove irrelevant or empty lanes. (**b**) Assessment of subcellular components in MACS purified and MACS + Percoll purified mitochondrial preparations. Markers used and predicted molecular weights: Na/K ATPase 112 kDa: plasma membrane, GRP78 78 kDa: endoplasmic reticulum, ATP5a 60 kDa: mitochondria, GAPDH 37 kDa: cytosol, Histone H3 17 kDa: Nucleus. Each panel represents an individual blot. BH = brain homogenate; mito = purified mitochondria. Molecular mass markers in kilodaltons are on the right. Each panel represents an individual blot.

All of the mitochondrial preparations were positive for PrP^C^ (Fig. 1a, top). Mitochondria from mice overexpressing PrP^C^ (Tg3F4 and RM) had the highest apparent relative abundances of PrP^C^, followed by C57BL/6 mice and Tg44 mice (Fig. 1a, top). The presence of PrP^C^ in mitochondria from Tg44 mice indicated that the GPI anchor was not required for PrP^C^ localization to the mitochondria. These data demonstrate that PrP^C^ is present in MACS purified mitochondria from young mice regardless of PrP^C^ expression level and that the GPI anchor is dispensable for PrP^C^ mitochondrial localization.

A known constituent of mitochondrial preparations is the ER-derived mitochondrial associated membrane (MAM). Although our preparations using the MACS protocol were highly enriched for mitochondria (Supplementary Fig. S1), it was possible that PrP^C^ was associated with either non-mitochondrial contaminants or was present in the MAM portion of our samples. We therefore further purified the mitochondria isolated using the MACS purification procedure by ultracentrifugation on a 30% Percoll cushion in order to remove any contaminating ER and MAM components. PrP^C^ was still detected by immunoblot albeit at reduced apparent concentrations compared to the standard MACS protocol (Fig. 1a, bottom). The decrease in PrP^C^ signal was likely due not only to the loss of MAMS but also to a decrease in the number of mitochondria isolated due to the extra purification steps. Nonetheless, a portion of the PrP^C^ did appear to remain associated with mitochondria isolated following centrifugation through a Percoll cushion.

To assess whether contamination from other subcellular components was present in our mitochondrial preparations, purified mitochondria were analyzed using a cocktail containing antibodies against various subcellular compartments and compared to the starting brain homogenate. Mitochondria purified with or without the Percoll cushion had undetectable levels of plasma membrane, nucleus, ER, and cytosol markers, suggesting a highly enriched mitochondrial preparation (Fig. 1b). Overall, these data strongly suggest that at least some PrP^C^ is present in brain derived mitochondria from wild-type C57BL/6 mice as well as transgenic mice expressing varying levels of human or mouse PrP^C^.

### LC/MS-MS analysis of isolated mitochondria

To confirm that PrP^C^ was present in isolated mitochondrial preparations, the protein content of MACS + Percoll purified mitochondria was analyzed using high resolution mass spectrometry. Many of the major proteins involved in oxidative phosphorylation were identified, confirming the presence of mitochondria. For example, the key mitochondrial marker cytochrome C oxidase IV (COXIV) was identified from 63 unique peptides and over 3,000 individual MS2 spectra in the C57BL/6 mouse mitochondrial preparation. Importantly, PrP^C^ peptides were detected in the mitochondria from all of the mice in this study. For example, the PrP peptide 208-VVEQMCVTQYQK-219 was identified and represents a common PrP peptide found in brain from prion infected and uninfected mice^36^,^37^,^38^,^39^,^40^. A complete list of the PrP peptides found in each of the MACS + Percoll purified mitochondrial preparations examined is shown in Table 1. N-terminal peptides of PrP^C^ were identified in WT, RM, and Tg3F4 mitochondria but we did not detect peptides corresponding to either the N-terminal signal peptide (1–23) or the C-terminal GPI signal peptide (229–254). These data are consistent with mitochondria-localized PrP^C^ being processed appropriately at the N and C terminus. Thus, the LC/MS-MS data both supported and confirmed the immunoblot detection of PrP^C^ in highly purified mitochondria.

### Mitochondrial PrP^C^ has mature N-linked glycans

**Figure 2:**
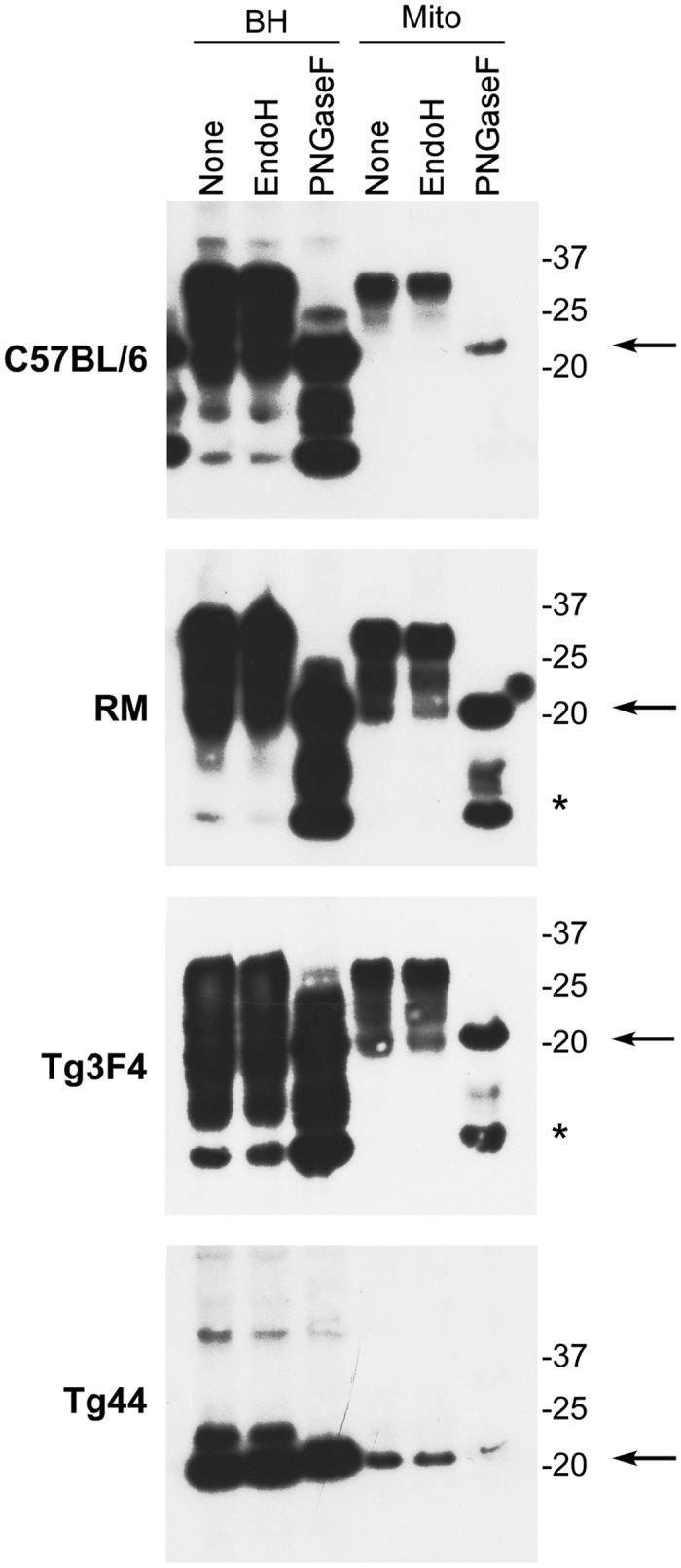
Mitochondrial PrP^C^ is fully glycosylated. Brain homogenate (10 μg) or MACS purified mitochondrial lysate (10 μg) was loaded into each lane. The rabbit monoclonal antibody EP1802Y, which recognizes a C-terminal epitope of PrP^C^, was used for detection. BH = brain homogenate; mito = MACS purified mitochondria. Molecular mass markers are shown on the right. The arrow indicates full length unglycosylated PrP^C^ while the asterisk indicates N-terminally truncated, unglycosylated PrP^C^. Each panel represents an individual blot. Blots were cropped for presentation purposes and to remove irrelevant or empty lanes.

The PrP^C^ sequence contains two potential sites for N-linked glycosylation at residues 181 and 197 and can exist in unglycosylated, mono-glycosylated, or di-glycosylated forms. All three glycoforms are typically detected in whole brain homogenates. To address whether mitochondrial PrP^C^ was fully processed with mature N-linked oligosaccharides similar to plasma membrane bound PrP^C^, we treated our samples with peptide-*N*-Glycosidase F (PNGaseF) or Endoglycosidase H (EndoH). Brain homogenate and mitochondrial preparations from RM, Tg3F4, and C57BL/6 mice were resistant to EndoH cleavage, indicating that mitochondrial PrP^C^ had been processed to complex glycans (Fig. 2). By contrast, they were sensitive to PNGaseF treatment similar to brain homogenate preparations and displayed a shift to a lower molecular weight consistent with cleavage of the N-linked glycans of PrP^C^ (Fig. 2, arrow). PrP^C^ associated with brain homogenates or with mitochondria from RM and Tg3F4 mice also showed a truncated band consistent with the presence of N-terminally truncated PrP^C^ (Fig. 2, asterisk). No such band was observed in the mitochondria from C57BL/6 or Tg44 mice which express lower levels of PrP^C^. Due to the very low levels of glycosylation in the anchorless PrP^C^ expressed in Tg44 mice, we could not determine whether mitochondrial PrP^C^ from Tg44 mice was sensitive to either EndoH or PNGaseF cleavage. Cumulatively, these data suggested that the N-linked glycans of mitochondrial PrP^C^ were mature and fully processed.

### Mitochondrial PrP^C^ is primarily localized to the inner mitochondrial membrane (IMM)

**Figure 3:**
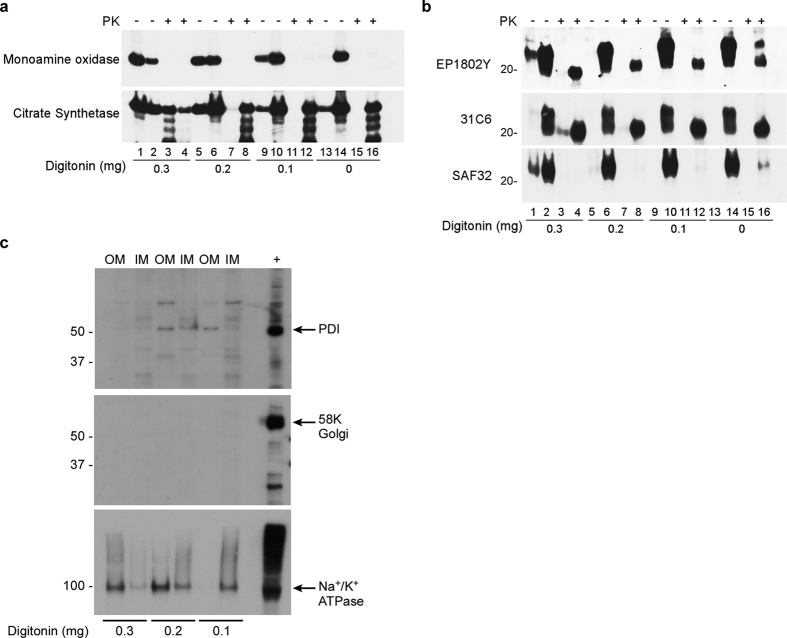
Mitochondrial PrP^C^ is associated primarily with the inner mitochondrial membrane/matrix fraction and is resistant to complete digestion by PK. For all panels odd numbered lanes are outer mitochondrial membrane (OMM) fractions and even numbered lanes are inner mitochondrial membrane/mitoplast (IMM or mitoplast) fractions. (**a**) Removal of the OMM with increasing concentrations of digitonin. The OMM marker monoamine oxidase and the matrix marker citrate synthetase were used to assess efficacy of digitonin treatment. Digestion with proteinase K (PK) is indicated above the gel. Each panel represents an individual blot. (**b**) Digestion of mitoplast with PK. PK treatment yields an ~20 kD C-terminal PrP^C^ fragment reactive with the C-terminal anti PrP antibodies EP1802Y (residues 222–226) and 31C6 (residues 143–149) while SAF32, which reacts with the N-terminal residues 58–88, showed limited reactivity. The 20 kD molecular mass marker is shown on the left. Each panel represents an individual blot. (**c**) Digitonin derived mitochondrial fractions were probed for the presence of ER (PDI), Golgi (58 K Golgi), and plasma membrane (Na^+^/K^+^ ATPase). Arrows identify the indicated protein in the brain homogenate positive control (+). Molecular weight markers are shown in kD on the left. OM = outer mitochondrial membrane; IM = inner mitochondrial membrane. Each panel represents an individual blot. All blots were cropped for presentation purposes and to remove irrelevant or empty lanes.

To determine where PrP^C^ was located within the mitochondrion, we treated mitochondria isolated from wild-type C57BL/6 mice with digitonin to strip away the outer mitochondrial membrane (OMM) from the inner mitochondrial membrane (IMM) and matrix (collectively known as mitoplast). Since removal of the OMM should supersede the need to remove MAM, we conducted our digitonin treatment on MACS purified mitochondria. As a control for the efficacy of digitonin treatment, we probed the OMM and mitoplast fractions with antibodies against the OMM protein monoamine oxidase (MAO) and the matrix protein citrate synthetase (CS) (Fig. 3a). Treatment of isolated mitochondria with increasing amounts of digitonin up to 0.3 mg resulted in more MAO being detected in the OMM fraction in a concentration dependent manner (Fig. 3a, top). Assessment of OMM and mitoplast fractions revealed that most of the mitochondria localized PrP^C^ was associated with the mitoplast fraction (Fig. 3b). However, we could not determine whether this association was absolute since we were unable to completely strip away the OMM from the mitoplast fraction without rupturing the resulting mitoplast as indicated by the presence of CS in both the OMM and mitoplast fractions with 0.3 mg of digitonin (Fig. 3a, bottom).

Proteins within the mitochondrial matrix or embedded in the inner mitochondrial membrane are protected from proteinase K (PK) digestion^41^,^42^. Thus, contingent upon the topological orientation of the molecule and its location on or within the mitoplast, treatment of mitoplasts with PK should either have no effect on PrP^C^, result in the loss of PrP^C^, or yield a partially PK-protected, truncated form of PrP^C^. To determine the orientation and sub-mitochondrial location of PrP^C^, OMM and IMM/mitoplast fractions were treated with PK following digitonin treatment. Treatment of OMM fractions with PK resulted in digestion of MAO while, consistent with its localization in the mitoplast, intact CS was more resistant to PK digestion (Fig. 3a). Thus, even in the absence of complete OMM removal, based on PK-sensitivity we could still differentiate between OMM and IMM proteins (Fig. 3a).

PrP^C^ was also undetectable in all OMM fractions except those treated with 0.3 mg of digitonin (Fig. 3b). By contrast, following digitonin and PK treatment, PrP^C^ associated with the mitoplast was partially protected by PK and shifted down in molecular weight to approximately 20 kD as detected by the rabbit monoclonal antibody EP1802Y (Fig. 3b top). Since EP1802Y reacts with a far C-terminal epitope (residues 220–131) in PrP^C^, we hypothesized that a portion of mitochondrial PrP^C^ was protected from PK digestion. To test this hypothesis, we probed digitonin derived, PK treated mitoplast fractions with the anti-PrP mouse monoclonal antibodies SAF32 and 31C6 which react with the octapeptide repeat region (residues 58–88) and residues 143–149, respectively. We observed no PrP^C^ reactivity with SAF32 while 31C6 showed reactivity similar to EP1802Y, suggesting that the octapeptide repeat region was absent from digitonin and PK-treated mitoplast associated PrP^C^ (Fig. 3b, middle and bottom).

To address the relative abundance of mitochondrial versus non-mitochondrial proteins in the digitonin derived OMM and IMM fractions, they were analyzed for their protein content using mass spectrometry. Peptides identified by Q-TOF LC-MS/MS of digitonin derived IMM mitochondrial fractions were ~93–97% mitochondrial in origin (Supplementary Fig. S2). The primary contaminants were cytoplasmic or nuclear in origin with some membrane proteins also detected (Supplementary Fig. S2). Cumulatively, these results support the notion that the IMM fraction from digitonin treated mitochondria is composed primarily of mitochondrial proteins and support our conclusion that the PrP^C^ identified within this fraction is unlikely to be a contaminant of the preparation.

**Figure 4:**
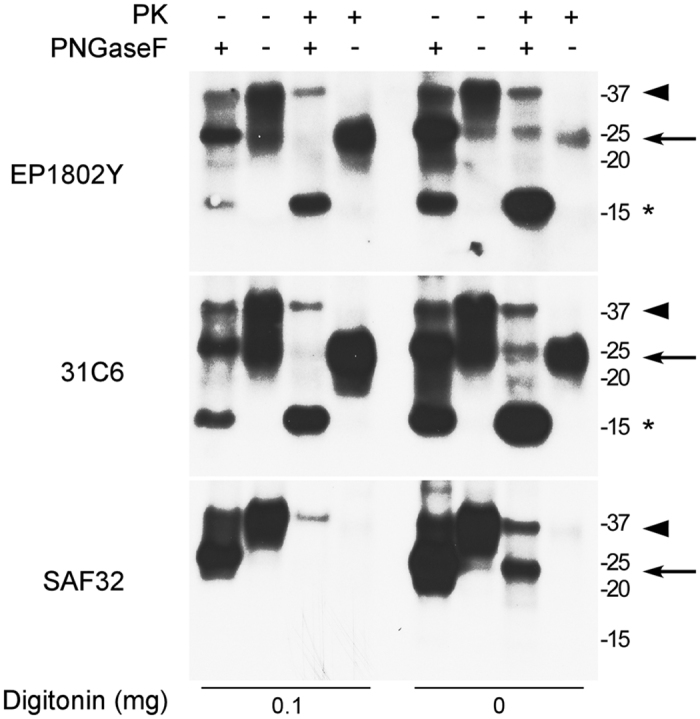
The N-terminus of fully glycosylated PrP^C^ in mitoplasts is PK-sensitive. PK digestion of inner mitochondrial membrane fractions (mitoplast) leads to a PrP^C^ band (arrow) which is not detected by the N-terminal PrP antibody SAF32. Following PNGaseF treatment, this band shifts to ~15 kD (asterisk). A non-PrP specific, cross-reactive band of approximately 36 kD is observed in all PNGaseF treated samples and may contribute to the intensity of the diglycosylated PrP bands (arrowhead). Molecular mass markers are shown on the right. Each panel represents an individual blot. Blots were cropped for presentation purposes and to remove irrelevant or empty lanes.

However, to further assess the presence of non-mitochondrial proteins in digitonin derived mitochondrial IMM and OMM sub-fractions, we also probed them for proteins associated with the most likely contaminating sources of PrP^C^: Na^+^/K^+^ ATPase (plasma membrane), protein disulfide isomerase or PDI (ER), and 58 K Golgi (the Golgi apparatus). Levels of both PDI and 58 K Golgi were negligible in all fractions. By contrast, Na^+^/K^+^ ATPase was detected in the IMM fractions indicating the presence of plasma membrane. However, with increasing digitonin concentrations it progressively moved into the OMM fraction (Fig. 3c). Importantly, the relative abundance of PrP^C^ in the IMM fraction did not change with increasing digitonin (Fig. 3b) supporting our hypothesis that PrP^C^ is indeed localized to the IMM and not a membrane contaminant. Consistent with the N-terminus of PrP^C^ being accessible to PK, treatment of digitonin derived PK-treated mitoplasts with PNGaseF to remove N-linked glycans resulted in collapse of the ~20 kD EP1802Y and 31C6 reactive bands to approximately ~15 kD (Fig. 4, arrow). No such band was detected using the SAF32 antibody. Overall, these results suggest that fully glycosylated PrP^C^ spans the IMM with the N-terminus through the octapeptide repeat region oriented toward the inner membrane space and the C-terminus facing the mitochondrial matrix.

### Co-localization of PrP^C^ and COXIV in mouse brain

**Figure 5:**
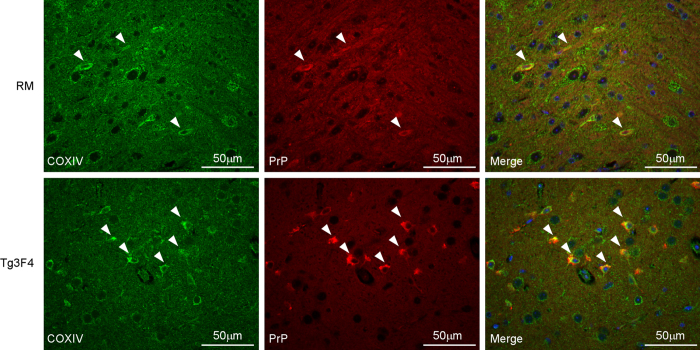
PrP^C^ co-localization with the mitochondrial protein COXIV is detectable in cells of the cortex in PrP^C^ overexpressing RM and Tg3F4 mice. Formalin fixed and paraffin embedded sagittal sections from the cortex were co-stained using a rabbit polyclonal antibody to COXIV (green, left panel) and the anti-PrP monoclonal antibody 3F4 conjugated to biotin (red, middle panel) as detailed in the methods. Merged images are shown in the panels on the right. White arrowheads indicate cells where COXIV and PrP^C^ co-localize. Scale bars are shown on the lower right. Magnification = 400X.

**Figure 6:**
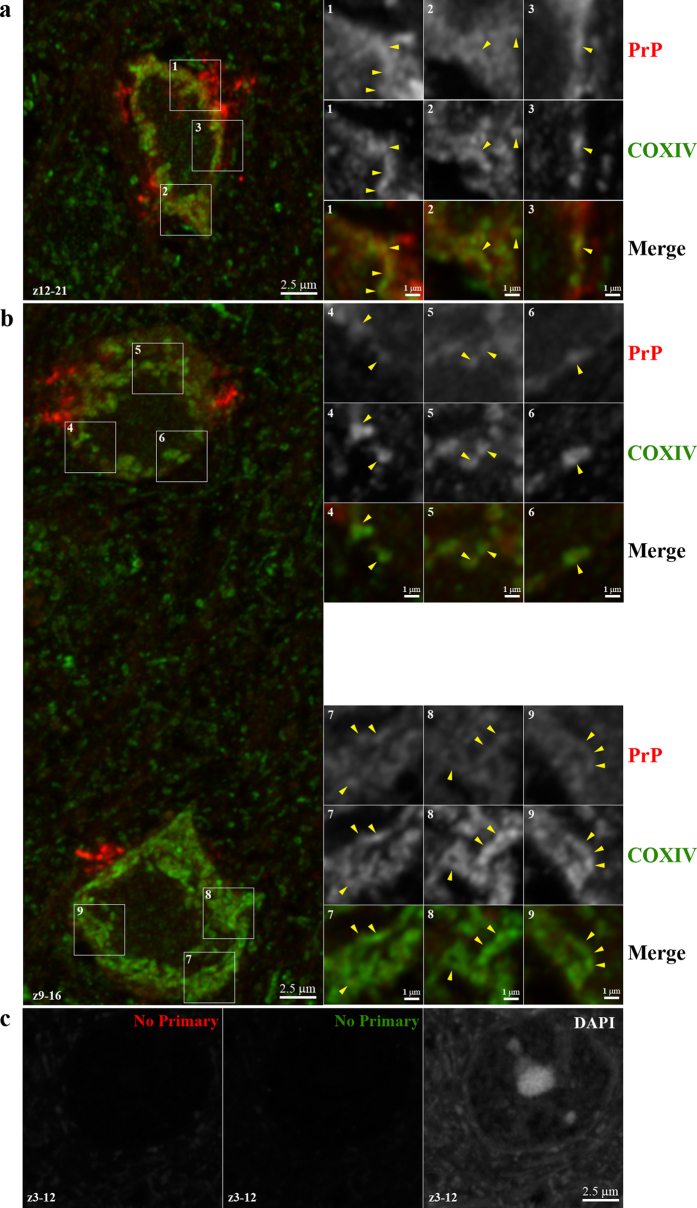
High resolution confocal microscopy of PrP^C^ co-localization with the mitochondrial protein COXIV. High-resolution confocal images were acquired from formalin fixed and paraffin embedded sagittal sections of cortex co-stained for COXIV and PrP^C^ as detailed in the Methods. Left-hand panels (**a** and **b**) show X-Y projections of confocal sections (0.16 mm each) from the subset of z-slices indicated in the lower left corner of each panel. Close-up images from a single z-slice of the boxed regions are shown on the right-hand. Yellow arrowheads indicate areas of co-localization. Lower panels (**c**) show no primary controls (Red = streptavidin conjugated AlexaFluor 568; Green = AlexaFluor 488) for the indicated z-slices and were acquired using the same settings as in panels (**a**) and (**b**). Scale bars are shown on the lower right.

We next addressed whether or not PrP^C^ was constitutively found in the mitochondria of all cells in the brain or only within a limited number of cells. Brain sections from 6–12 week old mice were examined by immunohistochemistry to determine whether PrP^C^ co-localized with the mitochondrial marker COXIV. In Tg3F4 and RM mice, both of which overexpress PrP^C^, we observed what appeared to be co-localization of PrP^C^ and COXIV in some cells, particularly in the cortex (Fig. 5). However, we were unable to detect clear PrP^C^ labeling in brain tissue from wild-type C57BL/6 and Tg44 mice which express a lower level of PrP^C^. To further address the potential localization of PrP^C^ to mitochondria in Tg3F4 mice, we performed high resolution confocal microscopy. PrP^C^ was clearly observed in the same focal plane as COXIV in cells of Tg3F4 mouse brain (Fig. 6 and Supplementary Fig. S3). Cumulatively, these results indicate that in at least 2 of our mouse models (RM and Tg3F4), PrP^C^ is detectable in brain mitochondria, suggesting that PrP^C^ does indeed localize to the mitochondrion in at least some cells.

### Localization of PrP^C^ to the IMM/mitochondrial matrix

**Figure 7:**
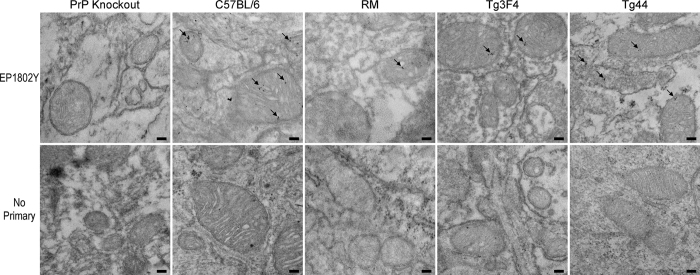
PrP^C^ in the mitochondria of RM, C57BL/6, Tg44, and Tg3F4 mice. Representative TEM images are shown. In all mice, the majority of immunogold particles are localized within the inner mitochondrial membrane and matrix. Mice lacking PrP (PrP knockout) showed no reactivity and no immunogold staining was detected in samples stained with the anti-rabbit secondary antibody alone (No primary). Arrows indicate a concentration of immunogold particles above background. All sections were probed with the anti-PrP rabbit polyclonal antibody EP1802Y followed by an anti-rabbit secondary antibody conjugated to 6 nm colloidal gold. Scale bar = 100 nM.

Transmission electron microscopy (TEM) was used to confirm the localization of PrP^C^ within the mitochondrion. Since our immunofluorescence study suggested that PrP^C^ staining was strongest in the cortex, we focused on this region. For TEM, cortical brain sections were probed with the anti-PrP^C^ rabbit monoclonal antibody EP1802Y followed by a secondary antibody conjugated to 6 nm gold particles. The gold particles were found associated with mitochondria in several cells of the posterior cortex from all mouse lines examined (Fig. 7 and Supplementary Fig. S4). Gold particles were primarily found associated with the IMM and matrix and were often found arranged in groups of 3 or more (Fig. 7 and Supplementary Fig. S4). These observations agree with most mitochondrial PrP^C^ being associated with the IMM of the mitoplast fraction as was observed following digitonin treatment (Fig. 3). PrP^C^ staining was also observed on double membranes consistent with either ER or plasma membrane (Supplementary Fig. S4). No PrP^C^ was detected in brain sections from PrP-knockout mice (Fig. 7 and Supplementary Fig. S4). Furthermore, exhaustive examination of negative control samples prepared without primary antibody were also negative for immunogold nanoparticles (Fig. 7, bottom row) indicating negligible non-specific secondary antibody reactivity. In agreement with our immunofluorescence findings, only some cells contained PrP^C^ positive mitochondria and not all mitochondria within that cell were positive for PrP^C^. Overall, the immunogold labeling of PrP^C^ and detection by TEM in multiple lines of young mice confirm and substantiate our finding that PrP^C^ is associated with mitochondria. Furthermore, these data support our conclusions from the PK protection study showing that mitochondrial PrP^C^ is associated primarily with the inner mitochondrial membrane and matrix.

## Discussion

Mitochondrial dysfunction is a hallmark of neurodegenerative disorders including Alzheimer’s and Parkinson’s disease^24^,^25^ and has been implicated in prion diseases^27^,^28^,^36^,^39^,^43^. Trafficking of PrP^C^ to the mitochondria has been associated with apoptosis^16^,^44^,^45^ suggesting a functional role for the localization of PrP^C^ to mitochondria. However, a clear association of PrP^C^ with mitochondria in the absence of overexpression has not been reported. We have found PrP^C^ in mitochondria isolated from the brains of young mice over-expressing human or mouse PrP^C^, underexpressing mouse GPI-anchorless PrP^C^, or expressing normal wild-type levels of mouse PrP^C^. The observed localization was independent of PrP^C^ expression level and species and was thus likely to be physiologically relevant. Our data are the first to demonstrate a physical interaction between PrP^C^ and the mitochondrion under homeostatic conditions and in the absence of disease, old age, or PrP^C^ mutations.

PrP^C^ associated with the mitochondria was in most respects indistinguishable from PrP^C^ normally associated with the plasma membrane. It was fully glycosylated and EndoH resistant with all major glycoforms present, and exhibited a similar pattern of N-terminal truncation as PrP^C^ from brain. However, the GPI anchor was not necessary for PrP^C^ localization to the mitochondrion and it differed from plasma membrane-anchored PrP^C^ in relation to its membrane environment and topological orientation. Wild-type PrP^C^ within the mitochondrion was largely resistant to extraction with digitonin, indicating that it was not localized to lipid rafts but rather to membrane domains with lower cholesterol content such as the IMM. Furthermore, unlike plasma membrane associated PrP^C^, it was also partially protected from protease digestion in isotonic buffers suggesting that it was not attached to the membrane via the GPI anchor but rather partially inserted into the membrane such that the C-terminus was protected. Taken together, these data strongly suggest that the PrP^C^ detected in brain mitochondria represents a population that is distinct from GPI-anchored PrP^C^ in the plasma membrane.

Our findings that mitochondrial PrP^C^ was partially protected from protease digestion and did not require the GPI anchor for mitochondrial localization (Fig. 1) suggested to us that it might be a membrane spanning isoform. It has been reported previously that PrP^C^ can exist in a number of topologically distinct isoforms with regard to how it is oriented within a membrane^19^,^21^. In particular, a transmembrane form of PrP^C^ where the C-terminus is on the inner side of the membrane (^CTM^PrP) has been observed in neuronal ER^17^,^21^ and Golgi^18^. Our observation that the C-terminus of mitoplast-associated PrP^C^ is protected from PK digestion while the N-terminus is not, suggests that mitochondrial PrP^C^ also has a transmembrane orientation. We hypothesize that PrP^C^ spans the inner mitochondrial membrane, most likely through the transmembrane forming hydrophobic stretch of amino acids from 110–135^21^,^46^, with the C-terminus residing in the mitochondrial matrix and the N-terminus facing the intermembrane space.

Immunohistochemical analysis looking at the co-localization of the mitochondrial protein COXIV and PrP^C^ in cortical cells (Figs 5 and 6) as well as TEM analysis of cortical brain tissue (Fig. 7 and Supplementary Fig. S4) clearly show that PrP^C^ is only occasionally found associated with mitochondria. By contrast, a previous survey of PrP^C^ in brain sections using Cryo-EM failed to identify clear PrP^C^ localization to mitochondria^14^. However, this is unsurprising as we only detected PrP^C^ in limited numbers of mitochondria in certain cells and survey methods using EM are highly inefficient due to the large magnifications involved. Our results suggest that PrP^C^ is not constitutively trafficked to mitochondria but may be localized to mitochondria only under certain cellular conditions or in certain cell populations. PrP^C^ localization to the mitochondria has been shown to correlate with neuronal apoptosis in aged FVB Tg(MoPrP)4053 mice which overexpress PrP^C^ 16, while Fas ligand engagement has been reported to induce PrP^C^ recruitment to mitochondria in T-lymphoblastoid CEM cells^44^. Other studies have shown that the PrP^C^ GPI-signal sequence (residues 232–254), if not degraded by the proteasome, localizes to mitochondria triggering depolarization and cellular apoptosis^47^,^48^. A similar phenomenon has been noted for the hydrophobic PrP^C^ 106–126 peptide which is highly toxic to cultured neurons^49^ and likely attacks the mitochondrial membrane resulting in depolarization and neuronal death^50^. These studies are all consistent with PrP^C^ associating with the mitochondrion only under certain conditions or during cellular stress. Thus, a direct PrP^C^-mitochondria association could help to explain the mitochondrial dysfunction observed during prion pathogenesis^27^,^28^. However, further work is needed to determine whether cells containing mitochondrial localized PrP^C^ are indeed stressed, apoptotic, or responding to some stimuli.

If PrP^C^ does indeed help to determine cell fate either through direct or indirect interaction with mitochondria or known mitochondrial pathways, the factor or factors it may be physically interacting with and the biochemical processes triggered are unknown. Our finding that the PrP^C^ N-terminus appears to be facing the inter membrane space is consistent with the possibility that mitochondrial PrP^C^ may play a role in transmitting specific cellular signals across the IMM. The N-terminus of PrP^C^ has been shown to bind many different molecules and contains an octapeptide repeat region that can bind metals, in particular copper^5^,^51^,^52^,^53^,^54^,^55^,^56^. Copper is a crucial cofactor for several mitochondrial proteins of the electron transport chain embedded within the IMM and Cu^2+^ is transported and kept ligand-bound within the mitochondrial matrix^57^,^58^,^59^. Thus, one possible role for PrP^C^ in the IMM is to control Cu^2+^ ingress and egress into and out of the mitochondrial matrix which may be altered under certain physiological conditions.

There have been a number of studies reporting on a correlation between PrP^C^ and Bax-mediated cellular apoptosis^6^,^60^,^61^,^62^,^63^,^64^,^65^,^66^,^67^,^68^,^69^,^70^, principally that PrP^C^ can inhibit Bax-mediated apoptosis^6^,^11^,^60^,^61^,^62^,^63^,^67^,^69^,^70^. However, a physical interaction between Bax and PrP^C^ has not been observed and the precise mechanism by which PrP^C^ interferes with Bax-mediated apoptosis has not been elucidated. Some studies have suggested that a cytosolic form of PrP is responsible for this^69^ and that α-helix 3 of PrP is necessary and sufficient for its anti-Bax function^70^. Since Bax must localize to the mitochondrion in order to execute its pro-apoptotic function^71^,^72^,^73^, the presence of PrP^C^ in the mitochondrion is consistent with the possibility that it may act to interfere directly or indirectly with Bax-mediated apoptosis.

Reports of N-glycosylated proteins within the mitochondria are extremely limited. One study identified a 45 kDa N-glycosylated protein in the inner mitochondrial membrane suggested to be a component of either NADH-ubiquinone oxidoreductase (complex I) or F_1_F_0_-ATPase (complex V)^74^. However, the question remains as to how N-glycosylated PrP^C^ is transported into the inner mitochondrial membrane and assumes a transmembrane orientation. It is known that PrP^C^ can assume a transmembrane orientation via the central hydrophobic region (109–132) and a recent study using HDX-MS showed that both wild-type and mouse PrP with the A116V mutation can form K^+^ and Ca^2+^ selective channels in lipid membranes^75^. Thus, a transmembrane orientation for PrP^C^ within the IMM is consistent with previous observations regarding the various ways PrP^C^ can interact with membranes. While the mechanism by which PrP^C^ may reach the mitochondria is unclear, it has been demonstrated that structural features within the N-termini of the PrP paralogs doppel and shadoo can target them to the mitochondrion under cellular stress conditions^76^, although the authors report that PrP is not targeted to mitochondria in such a manner. It is therefore likely that PrP^C^ targeting and import to the mitochondrion relies on another mechanism. A recent study demonstrated that PrP^C^ may form a ternary complex with the co-chaperone stress inducible protein 1/Hsp organizing protein (STIP1/HOP) and Hsp90^77^. Importantly, the chaperone proteins Hsp90 and Hsp70 play a critical role in the delivery of pre-proteins to the mitochondrial import receptor TOM70, which is principally responsible for signaling the import of proteins into the inner mitochondrial membrane^78^. Thus, we hypothesize that, under certain cellular conditions, PrP^C^ may be transported to TOM70 via an interaction with the Hsp70/Hsp90/HOP complex where it is then inserted into the IMM in a transmembrane orientation.

## Methods

### Mouse lines

The animal experimental protocol was reviewed and approved by the Rocky Mountain Laboratories Animal Care and Use Committee (Animal Study Protocol 2014-006). The Rocky Mountain Laboratories are fully accredited by the American Association for Laboratory Animal Care and this study was carried out in strict accordance with the recommendations in the Guide for the Care and Use of Laboratory Animals of the National Institutes of Health. The transgenic mouse lines used in this study do not express wild-type mouse PrP^C^ and have all been bred onto a C57BL/10 background and are characterized as follows. RM mice^79^ overexpress human PrP^C^ at approximately 4–8 times the normal level compared to wild-type mice. Tg3F4 mice (originally described as Tg(WT-E1^+/+^))^80^ lack native mouse PrP but overexpress mouse PrP with the 3F4 epitope at 4–8 times the level of wild-type mice. Tg44^+/+^ (referred to herein as “Tg44”) mice^81^ lack native mouse PrP and instead express mouse PrP with a stop codon preceding the GPI signal sequence that prevents GPI anchor addition. Anchorless PrP is expressed approximately 8 fold less than in wild-type mice^81^. PrP^O/O^(129/*Prn-p*^−/−^) mice^31^ do not express PrP and are also referred to as “PrP knockout”. The wild-type laboratory strain C57BL/6J is referred to herein as “C57BL/6”.

### Antibodies

The following anti-PrP antibodies were used: rabbit monoclonal EP1802Y (GeneTex) recognizing a C-terminal epitope of PrP^C^ (220–231)^82^, mouse monoclonal SAF32 to the octapeptide repeat region (residues 58–88) of both human and murine PrP, mouse monoclonal antibody 31C6^83^ (the kind gift of Dr. Motohiro Horiuchi, Hokkaido University, Japan) recognizing PrP residues 143–149, and mouse monoclonal 3F4 conjugated to biotin (Covance) which recognizes human PrP and residues 108 and 111 in mouse PrP with the 3F4 epitope. The secondary antibodies used were sheep anti-rabbit or anti-mouse IgG conjugated to horseradish peroxidase (GE Healthcare), streptavidin conjugated biotin linked to AlexaFluor 594 or 568, and goat anti-rabbit IgG conjugated to AlexaFluor 488, all from Molecular Probes. Goat anti-rabbit 6 nm gold conjugate was obtained from Electron Microscopy Sciences and the goat anti-rabbit 10 nm gold conjugate was obtained from BBI International.

The purity of the mitochondrial preparations was assessed using a cocktail of antibodies against common subcellular markers (Membrane Fraction WB Cocktail (Abcam)) according to the manufacturer’s instructions. The cocktail included antibodies reactive to the following subcellular proteins: Na/K ATPase 112 kDa: plasma membrane, GRP78 78 kDa: endoplasmic reticulum, ATP5a 60 kDa: mitochondria, GAPDH 37 kDa: cytosol, Histone H3 17 kDa: Nucleus. Rabbit polyclonal antibodies to the mitochondrial outer membrane marker monoamine oxidase (MAO, Sigma Aldrich, Atlas Antibodies) or the mitochondrial matrix markers citrate synthetase (CT, Abcam) and COXIV (ThermoScientific) were used to differentiate mitochondrial membrane fractions. Rabbit polyclonal antibody against protein disulfide isomerase (PDI, Cell Signaling), rabbit monoclonal antibody against 58 K Golgi (Genscript), and rabbit monoclonal antibody against Na^+^/K^+^ ATPase (Abcam) were used to assess levels of subcellular contamination in digitonin derived IMM and OMM fractions.

### Isolation of mitochondria

Brains from 6–12 week old wild-type C57BL/6 or transgenic mice were harvested and placed on ice. Each mitochondrial isolation was derived from 200 mg of starting brain tissue. The Miltenyi Mitochondria Extraction Kit-mouse (Miltenyi Biotech) and the Miltenyi Mitochondria Isolation Kit-mouse (Miltenyi Biotech) were used for tissue preparation and subsequent mitochondrial isolation. Miltenyi magnetic-activated cell sorting (MACS) was used to purify mitochondria from murine brain tissue according to the manufacturer’s instructions.

Selected samples of the MACS isolated mitochondria were further purified in order to separate the mitochondria from the endoplasmic reticulum (ER)-derived mitochondria associated membrane (MAM) using the method of Wieckowski *et al*.^84^ with minor modifications. These preparations are referred to as MACS + Percoll purified mitochondria. Briefly, 1 mL of MACS isolated mitochondria (~2 mg) were layered over 8 mL of Percoll medium ((225-mM mannitol, 25-mM HEPES pH7.4, 1 mM EGTA, and 30% Percoll (vol/vol)) in a 14 mL thin-wall polyallomar ultracentrifuge tube and 3.5 mL of mitochondria resuspension buffer (MRB, 250 mM mannitol, 5 mM HEPES pH 7.4, and 0.5 mM EGTA) was overlaid on top. Tubes were subjected to centrifugation at 95,000 *g* for 30 min at 4 °C. The MAM fraction formed a diffuse band approximately 1 cm from the top of the tube with mitochondria forming a dense band approximately 2 cm from the bottom of the tube. The separated mitochondrial fraction was washed and further prepared for immunoblotting and mass spectrometry as described below.

### PrP^C^ immunoblotting

Mitochondria isolated from C57BL/6, Tg3F4, RM, and Tg44 mice were lysed and denatured in a solution consisting of 4% SDS in modified mitochondrial resuspension buffer (MMRB, 250 mM sucrose, 15 mM KCL, 1 mM EGTA, 5 mM MgCl_2_, and 30 mM K_2_HPO4 pH 7.4). The samples were heated to 99 °C for 5 minutes and placed on ice for 2 minutes. Four volumes of ice-cold methanol were added to the lysates and samples held at −20 °C for approximately 12 hours. Samples were centrifuged at 20,000 *g* for 15 minutes and the resulting pellets were resuspended in 50 μL of 4X lithium dodecyl sulfate sample buffer (LDS, Life Technologies) containing 2.5 μL of 14.3 M β-mercaptoethanol. Approximately 10 μg of mitochondrial protein lysate from each mouse sample was loaded onto 4–12% NuPAGE gels (Life Technologies) then run at 160 V for 55 minutes in 2-(N-morpholino)ethanesulfonic acid (MES) running buffer (Life Technologies). Gels were blotted onto polyvinylidene difluoride (PVDF) membranes at 90 V for 75 minutes and the membrane was blocked by incubation with 5% powdered milk in TBST (10 mM Tris pH 8.0, 150 mM NaCl, 0.05% Tween-20) for 1 hour. The rabbit polyclonal anti-PrP antibody EP1802Y was used diluted 1:2500 in TBST as described above and then incubated with the membrane overnight with rocking at 4 °C. The membrane was washed 6 times for 5 minutes each with gentle agitation and a sheep anti-rabbit HRP conjugated secondary antibody diluted 1:6500 in TBST was incubated with the membrane for 2 hours at room temperature with gentle agitation. Following 6 × 5 minute washes, protein was detected with ECL Prime (GE Healthcare) according to the manufacturer’s instructions.

### Immunofluorescent immunohistochemistry

Formalin fixed and paraffin embedded sagittal sections of mouse brain (5 microns) were deparaffinized and rehydrated followed by antigen retrieval and blocking as described previously^85^. All subsequent manipulations were done at room temperature unless otherwise indicated. Sections were incubated for 1 hr with a rabbit polyclonal COXIV antibody diluted 1:100 in 1X phosphate buffered saline (PBS)/1% donkey serum (DS, Invitrogen) followed by 2 × 10 min rinses in 1X PBS/0.05% fish skin gelatin (FSG, Sigma-Aldrich). Slides were then incubated in goat anti-rabbit IgG conjugated to AlexaFluor 488 antibody diluted 1: 500 in 1X PBS/1% DS for 1 hour, rinsed 2 × 10 mins in 1X PBS/0.05% FSG and blocked as described previously^85^. Slides were then incubated at 4 °C overnight with the mouse monoclonal antibody 3F4 conjugated to biotin (1:50 dilution), rinsed 2 × 10 mins, incubated with a 1:500 dilution of streptavidin conjugated to AlexaFluor 594, and rinsed again for 2 × 10 mins as described above. Coverslips were mounted using the ProLong Gold antifade agent plus DAPI (Molecular Probes). Images were captured using a Nikon Digital Sight camera and NIS elements.

Sections analyzed using confocal microscopy were processed the same way except that samples were incubated with the COXIV antibody overnight at 4 °C prior to development with AlexaFluor 488 and incubated for 1 hour at room temperature with biotinylated 3F4 prior to development with AlexaFluor 568 conjugated to streptavidin. High-resolution confocal images were acquired with a Zeiss LSM 880 Airyscan microscope using a 63x/1.40 Plan-Apochromat Oil DIC M27 objective lens (Carl Zeiss Microscopy). Z-stacks of entire cells were acquired with 0.16 mm intervals and processed using Zen image acquisition and processing software (Zen 2.3 version 13, 64 bit; Carl Zeiss Microscopy). Images shown in figures were cropped and adjustments to brightness and contrast made using Fiji^86^ and Adobe Photoshop CS6 software (Adobe Systems).

### PNGaseF and EndoH treatment of isolated mitochondria

To address whether mitochondrial PrP^C^ was fully processed with mature N-linked oligosaccharides, samples were treated with peptide-*N*-Glycosidase F (PNGaseF) (New England Biolabs) and Endoglycosidase H (EndoH) (New England Biolabs). Protein was precipitated from brain homogenates and MACS purified mitochondria by methanol precipitation as described above. PNGaseF and EndoH treatment were performed according to the manufacturer’s suggested reaction conditions. Immunoblotting for PrP^C^ was performed as described above with the EP1802Y antibody.

### Proteinase K protection assay and digitonin sub-fractionation of mitochondria

Purified mitochondria (5 mg) were resuspended in 200 μL of ice cold isotonic KCl^-^ buffer (0.18 M KCl, 1 mM EDTA, 5 mM MOPS pH 7.25)^87^ containing 0, 0.1, 0.2, or 0.3 mg of digitonin. Samples were rotated at 4 °C for 15 minutes followed by centrifugation at 12,000× *g* for 10 minutes. The supernatant, composed of the outer mitochondrial membrane (OMM) and associated proteins was removed and transferred to a clean tube, leaving the inner mitochondrial membrane (IMM) and matrix fraction (mitoplast) as a pellet. The mitoplasts were resuspended in 200 μL of isotonic KCl^-^ buffer and the OMM and mitoplast fractions were prepared for immunoblot as described above. One portion of the OMM and IMM fractions was treated with 0.3 μg/mL proteinase K at 37 °C for 30 minutes while another was taken additionally for PNGaseF treatment as described above. To assess the efficacy of digitonin solubilization of the OMM, fractions were probed with primary antibody against either the OMM MAO (diluted 1:2,500) or the IMM marker CS (diluted 1:2,500). PrP^C^ was detected using the primary antibodies EP1802Y, SAF-32, or 31C6 all diluted 1:1,500. Antibodies against marker proteins for the ER, Golgi, and plasma membrane were diluted at 1:15,000 and used to assess the levels of these proteins in digitonin fractionated mitochondria. A goat secondary anti-rabbit IgG was used at a dilution of 1:30,000. Protein preparation and immunblotting were carried out as described above.

### Transmission electron microscopy (TEM)

Necropsied tissue fragments from the posterior cerebral cortex of mice perfused with fixative containing 4% paraformaldehyde and 0.1% glutaraldehyde in Sorenson’s phosphate buffer (Electron Microscopy Sciences, Hatfield, PA) were cut into pieces approximately 1 mm^3^ and immersed into fresh 1 mL volumes of fixative. The samples were processed for immune electron microscopy using a model Biowave microwave and ColdSpot system (Ted Pella, Inc., Redding, CA) at 22–24 °C as follows. Primary fixation was completed with 2 power cycles of 2 min on, 2 min off, and 2 min on at 170 W (2-2-2). Samples were washed with two changes of phosphate buffer (pH 7) for 2 min each, then post-fixed with 0.1% osmium tetroxide and 0.08% potassium ferrocyanide in Sorrenson’s phosphate buffer (pH 7) for 2 cycles of 2-2-2 (see above). The tissues were washed twice with deionized water for 2 min each, stained with 1% filtered aqueous uranyl acetate, using 2 cycles of 2-2-2. The samples were washed twice for 2 min in water, and dehydrated for 2 min each at 250 W with 70%, 100%, and 100% ethanol. The samples were then infiltrated with araldite resin (SPI Supplies, Inc.,) at 250 W for 2 × 10 min in 50% araldite/ethanol, and twice for 2 × 20 min in 75% araldite/ethanol, and 2 × 40 min in 100% araldite. The samples were placed into flat embedding molds and polymerized overnight at 65 °C. Silver sections were cut with a diamond knife and mounted on 200 mesh gold grids. To assure adherence of sections, grids were heated to 60 °C for 2 hr.

Immunolabeling of sections was conducted using microwave irradiation at 170 W and 22–24 °C as follows. All steps were performed in 20 μL droplets placed in wells of immunostaining pads (Ted Pella, Inc.) with microwave irradiation. Saturated humidity was maintained by placing the immunostaining pad onto a water-soaked gauze sponge placed within a 100 mm plastic petri dish. Sections were etched for 1 min in 10% aqueous sodium metaperiodate (Sigma-Aldrich Chemical) and washed twice for 1 min with water. Antigen retrieval was performed by treating with 70% formic acid for 1 min, followed by two 1 min water washes. The grids were transferred to droplets of blocking buffer containing 2% globulin-free bovine serum albumin (Sigma-Aldrich) in phosphate buffered saline (BSA-PBS) for 2 min. Grids were incubated in a 1:100 dilution of EP1802Y primary antibody in BSA-PBS or buffer alone for either 2 cycles of 2-2-2, or overnight without irradiation at 4 °C. Samples were washed twice in BSA-PBS with microwave irradiation for 1 min, and incubated in a 1:100 dilution of secondary anti-rabbit Ab-colloidal gold (6 nm) for 2 cycles of 2-2-2. The labeled grids were washed twice for 1 min in BSA-PBS, twice for 1 min in PBS, and 3 times for 1 min in filtered deionized water, wicked with filter paper and dried. All grids were examined at 80 kV on a model H-7500 transmission electron microscope (Hitachi High Technologies). Images were captured and recorded using a model HR-100 CCD camera and software (Advanced Microscopy Techniques).

### Cryo-electron microscopy

Mitochondrial pellets fixed with 0.1% glutaraldehyde/4% paraformaldehyde in buffer were washed briefly with Hank’s balanced salt solution (HBSS) and embedded in 10% gelatin. After 1 hour at 4 °C, the gelatin was cut into small cubes and placed overnight at 4 °C in 2.3 M sucrose for infusion. Cubes were frozen and trimmed at −105 °C and sectioned at −120 °C. Sections of approximately 70 nm were picked up with a mixture of 2% methyl cellulose (MC) in water/2.3 M sucrose in 0.1 M phosphate buffer (PB) and placed on 200 mesh formvar/carbon coated nickel grids. Immunology was performed as follows: Grids were incubated in 0.1% glycine in PB for 5 minutes and then blocked with 1% bovine serum albumin/Tris 7.2 for 15 minutes. Grids were placed on droplets of primary antibodies diluted 1:30 for 30 minutes. After 4 × 2 minute washes with blocking buffer, the grids were placed on droplets of 10 nm gold conjugates diluted 1:25 in the blocking solution for 30 minutes. Grids were then washed in PBS for 4 × 2 minutes followed by 6 × 1 minute dH2O washes. Grids were passed over 2 drops of 0.4% uranyl acetate/1.8% MC pH 7.2 and after 10 minutes of floating were picked up and allowed to dry prior to examination on a Tecnai BT Spirit electron microscope at 120 kV (FEI).

### Orbitrap high resolution mass spectrometry

Protein from MACS + Percoll purified C57BL/6, Tg3F4, RM, and Tg44 mitochondria were lysed and precipitated as described above. Mitochondrial protein pellets were isolated by centrifugation and further denatured with a membrane solubilization buffer (7 M urea, 2 M thiourea, 1% 3-(4-Heptyl)phenyl-3-hydroxypropyl)dimethylammoniopropanesulfonate (C_7_BzO, Sigma-Aldrich) with 15 mM dithiothreitol (DTT) and heated at 37 °C at 300 rpm on an Eppendorf Thermomixer for 30 min prior to alkylation with 75 mM iodoacetamide (ThermoFisher) for 30 min at room temperature in the dark with gentle rotation. Excess iodoacetamide was quenched by the addition of dithiothreitol (DTT) from a 1 M stock solution to a final concentration of 90 mM. Sample buffer containing lithium dodecyl sulfate (LDS) was added and the resulting solution (~50 μL) was boiled for 2 min, then subjected to electrophoresis using NuPAGE 10% Bis-Tris 1.5 mm gels with MES running buffer at 175 V. Gels were then stained with Coomassie blue Imperial stain (ThermoFisher) and destained overnight with water. The entire lane was excised from the gel into 16 slices using a razor blade and the bands were then subjected to tryptic digestion in 50 mM ammonium bicarbonate, pH 8 with 10% trifluoroethanol for 16 hours at 37 °C. The tryptic peptides were then collected in fresh Protein LoBind tubes (Eppendorf) and the gel slices were further triturated with 70% acetonitrile (ACN) in H_2_O. These solutions were then concentrated by speed-vac to dryness in 200 uL polypropylene auto-sampler vials (Sun Sri) and kept at −20 °C until analyzed.

Trypsin-digested peptides were suspended in 10 μL of Buffer A (0.1% formic acid (FA), 2%ACN, and 98% H_2_O) and subjected to chromatography using a Reprosil Pure C18 reverse phase media (3 um particle size and 120 A pore) packed in a pulled tip, nano-chromatography column (0.075 mm ID × 350 mm) from Precision Capillary Columns, Inc. Peptides were separated at 200 nL/min using a ProXeon nLC-1000 multi-dimensional liquid chromatograph equipped with an Ion Max Nanospray source (ThermoFisher Scientific) in-line with a Fusion Orbitrap mass spectrometer (ThermoFisher Scientific) under the control of Xcalibur 3.0 software used for data acquisition. The mobile phase consisted of a linear gradient prepared from solvent A and solvent B (0.1% FA, 2% water, and 98% ACN) at room temperature. Mass calibration was performed with the positive ion Cal Mix (ThermoFisher) and monitored by routine analysis of a 10 fmol aliquot of BSA tryptic digest. Searches for the BSA runs were conducted using MASCOT v2.5.1 search engine, typically yielding a MASCOT protein score of 2,000–5,000 with 75–85% coverage and within 4 ppm mass error using a Swiss-Prot database and Proteome Discoverer 2.0 for data visualization.

Raw files from the LC-MS/MS runs were searched using PEAKS DB Studio v7.5^88^. Monoisotopic parent mass ion tolerances were set to 25 ppm and MS/MS fragment tolerances were set to 0.6 Da with a maximum number of 2 possible missed tryptic cleavages. The data were searched against a Swiss-prot database with mouse taxonomy consisting of 16,306 unique protein sequences. The RM mice express PrP^C^ with human sequence and the Tg3F4 mice express PrP^C^ with a 3F4 epitope in which mouse residues lysine and alanine at residues 108 and 111, respectively, are both replaced by methionine. In order to reflect these changes, separate Swiss-prot mouse databases were constructed for this study. All other entries for mouse proteins were unchanged. Cysteine was assigned a fixed carbamidomethyl modification of +57.02 and methionine a variable modification of +15.99 for the potential presence of sulfoxide residues. The search results were set to a 0.1% false discovery rate (FDR) and at least 2 peptides with unique m/z values and −10 log P score threshold of >20 were required for a positive protein identification.

### Q-TOF mass spectrometry

Mitochondrial inner and outer membrane samples were subjected to SDS-PAGE with Commassie blue staining. Each lane was excised into 10 gel slices and subjected to in-gel digestion with a trypsin\Lys-C mixture as described above. Individual digests were then analyzed by LC–MS/MS using an Agilent 1200 series nanoflow HPLC equipped with a chip-cube nanospray source connected to an Agilent 6550 iFunnel Quadrupole Time-of-Flight (Q-TOF) system (Agilent Technologies). Samples were loaded onto a 40 nL trapping column using 3% ACN/0.1% FA and then separated on a 75 μm × 43 mm analytical column (Agilent G4240-62005-ZORBAX 300SB-C18). Separation was achieved with a reversed phase step gradient at 0.6 μL/min starting with 97% Buffer A (H_2_O/0.1%FA) to 20% Buffer B (H_2_O/90%ACN/0.1%FA) in 20 minutes, then to 40% B in 32 minutes, 90% B in 35 minutes, and then held at 90% B from 35 to 40 minutes and back to 97% A by 42 minutes. The survey scan was done with positive ion polarity with an *m*/*z* range from 300–1700 *m*/*z* at a scan rate of 4 spectra/second and an MS/MS scan rate of 2 spectra/second with a maximum of 20 precursor ions selected for MS/MS per cycle.

## Additional Information

**How to cite this article**: Faris, R. *et al*. Cellular prion protein is present in mitochondria of healthy mice. *Sci. Rep.* **7**, 41556; doi: 10.1038/srep41556 (2017).

**Publisher's note:** Springer Nature remains neutral with regard to jurisdictional claims in published maps and institutional affiliations.

## Supplementary Material

Supplementary Information

## Figures and Tables

**Table 1:** PrP peptides found in MACS + Percoll purified mitochondria by high resolution LC-MS/MS.

Mouse line	PrP peptide^a^	Residues	Spectral Counts^b^	Mass^c^	*m*/*z*^d^	ppm^e^
C57BL/6	YPGQGSPGGNR	38–48	1	1088.5	545.255	−3.4
	VVEQ**M**CVTQYQK	208–219	7	1527.706	764.858	−2.8
	ESQAYYDGR	220–228	2	1087.457	544.735	−2.3
RM	PGGWNTGGSR	28–37	3	987.4522	494.733	−0.6
	YPGQGSPGGNR	38–48	77	1088.5	545.256	−2.2
	PIIHFGSDYEDR	137–148	22	1447.673	483.564	−2.7
	YPNQVYYR	157–164	10	1101.524	551.768	−2.6
	GENFTETDVK	195–204	5	1138.514	570.262	−3.8
	VVEQ**M**CITQYER	209–220	13	1570.712	786.360	−3.7
	ESQAYYQR	221–228	27	1043.467	522.740	−1.4
Tg3F4	PGGWNTGGSR	28–37	1	987.4522	494.734	1.5
	YPGQGSPGGNR.Y	38–48	18	1088.5	545.257	−0.2
	H**M**AGAAAAGAVVGGLGGYMLGSA**M**SR	110–135	1	2394.124	799.048	−0.7
	PMIHFGNDWEDR	136–147	3	1515.657	506.225	−1.7
	P**M**IHFGNDWEDR	136–147	3	1531.651	511.557	−0.9
	YPNQVYYR	156–163	5	1101.524	551.769	−1.2
	GENFTETDVK	194–203	11	1138.514	570.264	0
	VVEQMCVTQYQK	208–219	19	1511.711	756.862	−1.1
	VVEQ**M**CVTQYQK	208–219	17	1527.706	764.86	0
	ESQAYYDGR	220–228	16	1087.457	544.736	0.1
Tg44	GENFTETDVK	194–203	4	1138.514	570.265	0.9
	VVEQMCVTQYQK	209–219	1	1511.711	756.863	−0.1
	VVEQ**M**CVTQYQK	208–219	1	1527.706	764.86	−0.1
	ESQAYYDGR	220–228	3	1087.457	544.736	0

^a^Samples were analyzed using a high resolution Fusion Orbitrap mass spectrometer. Peptides with unique observed mass over charge (*m*/*z*) values identified by PEAKS DB search engine with a 0.1% FDR and a −10 logP threshold value of >30. Oxidized methionine residues are shown in bold.

^b^The number of assigned tandem mass spectra or spectral counts associated with that peptide.

^c^The calculated monoisotopic mass of the intact peptide.

^d^Experimentally observed *m*/*z* value of the precursor ion.

^e^Calculated error in parts per million of the experimentally observed monoisotopic peptide *m*/*z* value.
