# Supplementary material for: Cellular prion protein is present in mitochondria of healthy mice
Source: Sci Rep. 2017 Feb 2;7:41556. doi: 10.1038/srep41556 (PMC5288712; doi:10.1038/srep41556)
Supplement: Supplementary Information [file srep41556-s1.pdf]

Supplementary Information

Cellular prion protein is present in mitochondria of healthy mice

Robert Faris[1], Roger A. Moore[1], Anne Ward[1], Brent Race[1], David W. Dorward[2], Jason R. Hollister, Elizabeth R. Fischer, and Suzette A. Priola*[1]

[1]Laboratory of Persistent Viral Diseases and [2]Research Technologies Branch, Rocky Mountain Laboratories, National Institute of Allergy and Infectious Diseases, Hamilton, Montana 59840

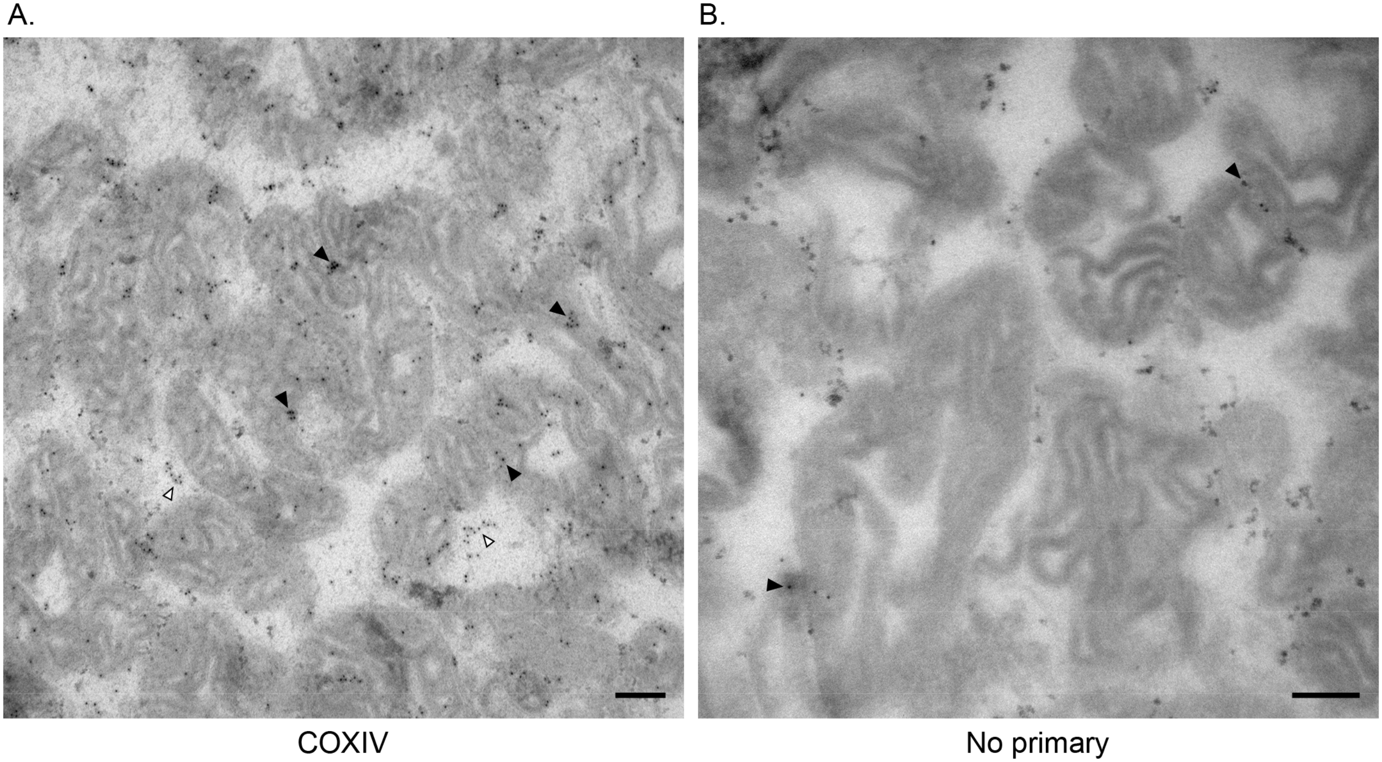

Supplementary Figure S1. Immunogold labeling of MACS isolated mitochondrial preparations with COXIV. MACS isolated mitochondria were prepared for Cryo-EM and stained with a rabbit polyclonal antibody to the mitochondrial inner membrane marker COXIV followed by an anti-rabbit gold conjugated secondary antibody as described in the Methods. Nearly all observable mitochondrial structures showed immunogold labeling (A) while little or no labeling was seen in samples developed with secondary antibody only (B).

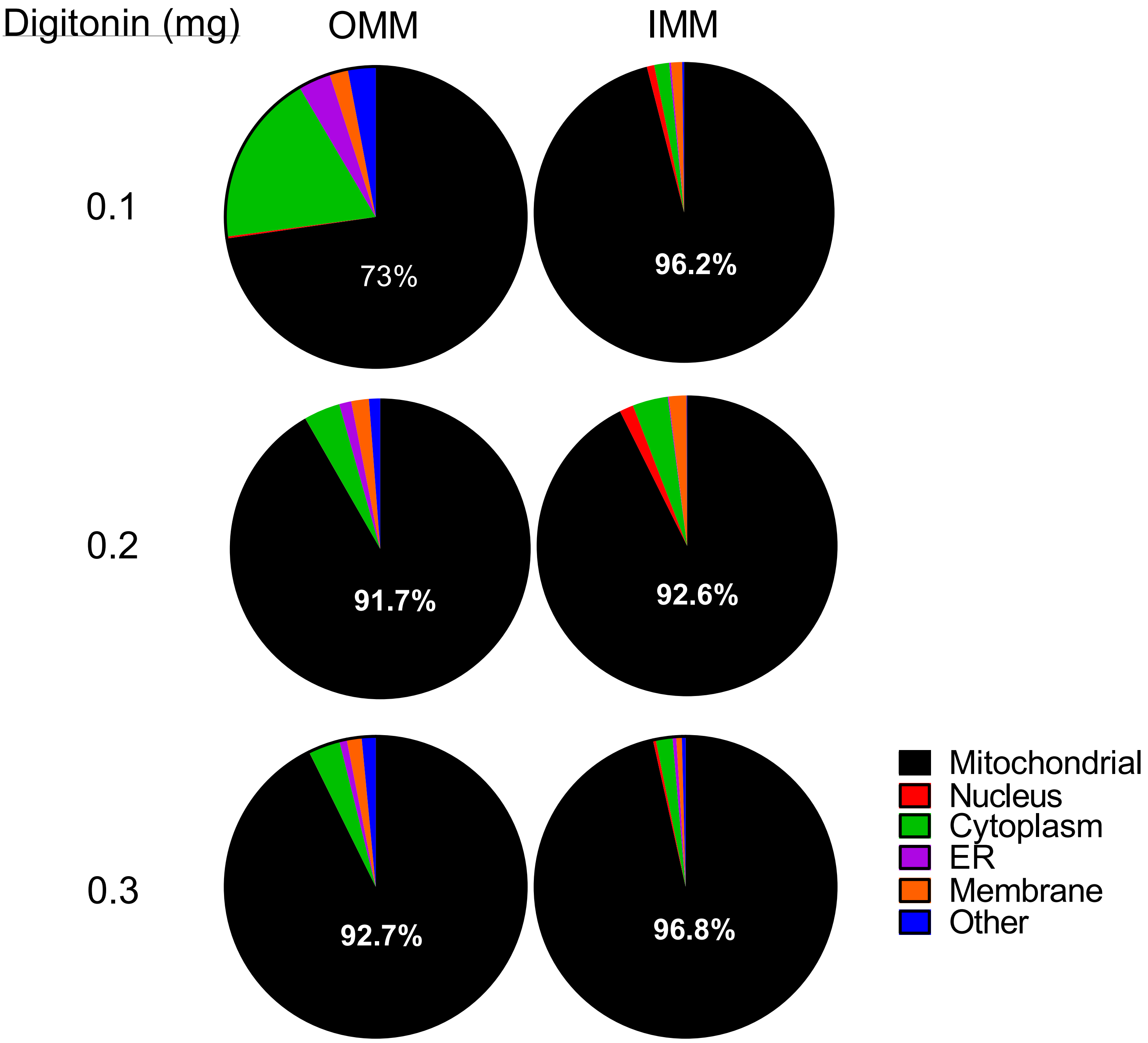

Supplementary Figure S2. Digitonin derived IMM and OMM mitochondrial fractions contain primarily mitochondrial proteins. Digitonin derived IMM and OMM fractions were assayed for their protein content by LC-MS/MS. Data was filtered for mitochondrial peptides by searching against the MitoCarta database coupled with manual confirmation of protein localization via Uniprot. Peptides from non-mitochondrial proteins comprised between 3-7% of the total peptides identified in the IMM fractions. Samples were analyzed using an Agilent 6550 Series Accurate-Mass Quadrupole Time-of-Flight (Q-TOF) LC-MS/MS system as described in the Methods.

Box 3 (z21) Box 6 (z16)

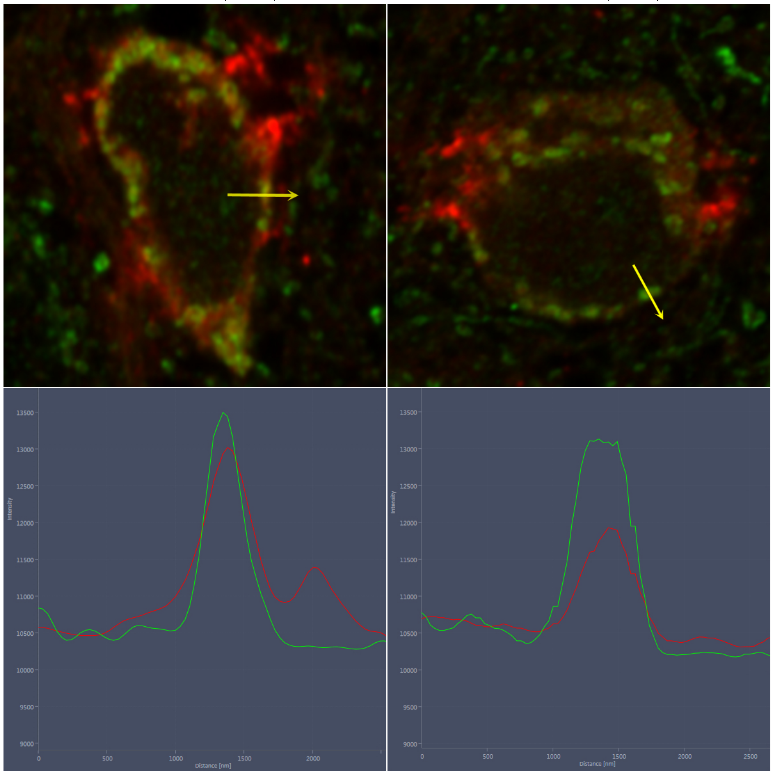

Supplementary Figure S3. Co-localization of COXIV and PrP^C in cells of the cortex. Cell images are from a single slice of the z-stack images shown in Figure 6a and b with COXIV staining in green and PrP^C staining in red. The z-slice number is indicated in parentheses. The yellow arrows are drawn through an ~2.5 µm segment from Box 3 in Fig. 6a and from Box 6 in Fig. 6b. The arrows correspond to the fluorograms shown in the bottom panels. There is a clear and coincident increase in fluorescent intensity for both COXIV (green line) and PrP^C (red line) in each fluorogram, indicating an area of the cell with close co-localization of the two molecules.

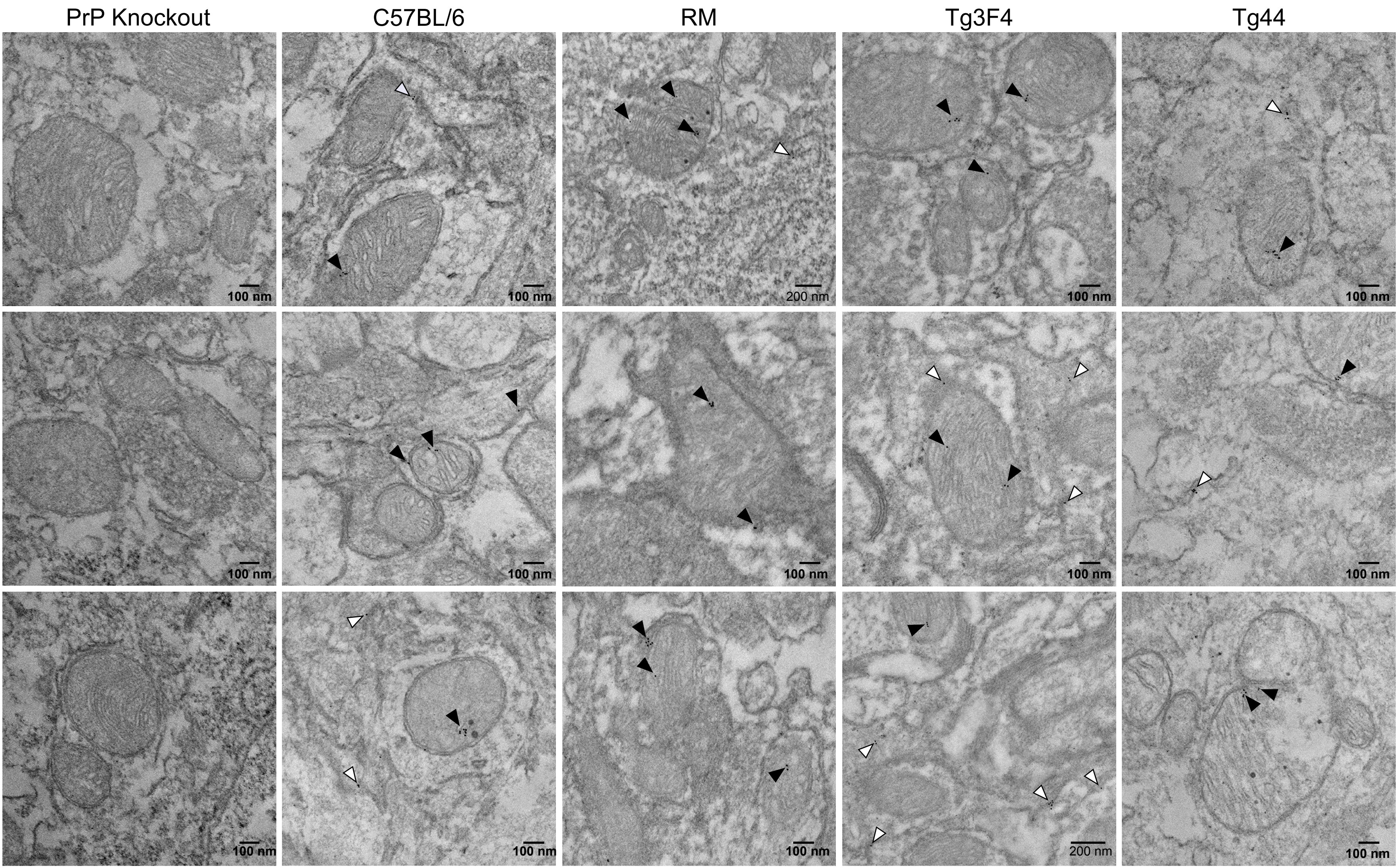

Supplementary Figure S4. Immunogold labeling of PrP^C in mouse brain. Brain sections from PrP^C knockout, C57BL/6, RM, Tg3F4 and Tg44 mice were stained with the anti-PrP rabbit monoclonal antibody EP1802Y and developed for TEM as described in the methods. The majority of immunogold particles were localized over the inner mitochondrial membrane and matrix (black arrowheads). Additional PrP^C staining was observed over double membranes consistent with ER or plasma membrane (white arrowheads). No immunogold staining was detected in PrP^C knockout mice.
